# Induction of autophagy by ARHI (DIRAS3) alters fundamental metabolic pathways in ovarian cancer models

**DOI:** 10.1186/s12885-016-2850-8

**Published:** 2016-10-26

**Authors:** Argentina Ornelas, Christopher R. McCullough, Zhen Lu, Niki M. Zacharias, Lindsay E. Kelderhouse, Joshua Gray, Hailing Yang, Brian J. Engel, Yan Wang, Weiqun Mao, Margie N. Sutton, Pratip K. Bhattacharya, Robert C. Bast, Steven W. Millward

**Affiliations:** 1 Department of Cancer Systems Imaging, the University of Texas M.D. Anderson Cancer Center, Houston, USA; 2 Department of Experimental Therapeutics, the University of Texas M.D. Anderson Cancer Center, Houston, USA; 3 Department of Bioengineering, Rice University, Houston, USA

**Keywords:** ARHI, Autophagy, Metabolism, Glutaminolysis, Ovarian cancer, NMR, Necroptosis

## Abstract

**Background:**

Autophagy is a bulk catabolic process that modulates tumorigenesis, therapeutic resistance, and dormancy. The tumor suppressor ARHI (DIRAS3) is a potent inducer of autophagy and its expression results in necroptotic cell death in vitro and tumor dormancy in vivo. ARHI is down-regulated or lost in over 60 % of primary ovarian tumors yet is dramatically up-regulated in metastatic disease. The metabolic changes that occur during ARHI induction and their role in modulating death and dormancy are unknown.

**Methods:**

We employed Nuclear Magnetic Resonance (NMR)-based metabolomic strategies to characterize changes in key metabolic pathways in both cell culture and xenograft models of ARHI expression and autophagy. These pathways were further interrogated by cell-based immunofluorescence imaging, tracer uptake studies, targeted metabolic inhibition, and in vivo PET/CT imaging.

**Results:**

Induction of ARHI in cell culture models resulted in an autophagy-dependent increase in lactate production along with increased glucose uptake and enhanced sensitivity to glycolytic inhibitors. Increased uptake of glutamine was also dependent on autophagy and dramatically sensitized cultured ARHI-expressing ovarian cancer cell lines to glutaminase inhibition. Induction of ARHI resulted in a reduction in mitochondrial respiration, decreased mitochondrial membrane potential, and decreased Tom20 staining suggesting an ARHI-dependent loss of mitochondrial function. ARHI induction in mouse xenograft models resulted in an increase in free amino acids, a transient increase in [^18^F]-FDG uptake, and significantly altered choline metabolism.

**Conclusions:**

ARHI expression has previously been shown to trigger autophagy-associated necroptosis in cell culture. In this study, we have demonstrated that ARHI expression results in decreased cellular ATP/ADP, increased oxidative stress, and decreased mitochondrial function. While this bioenergetic shock is consistent with programmed necrosis, our data indicates that the accompanying up-regulation of glycolysis and glutaminolysis is autophagy-dependent and serves to support cell viability rather than facilitate necroptotic cell death. While the mechanistic basis for metabolic up-regulation following ARHI induction is unknown, our preliminary data suggest that decreased mitochondrial function and increased metabolic demand may play a role. These alterations in fundamental metabolic pathways during autophagy-associated necroptosis may provide the basis for new therapeutic strategies for the treatment of dormant ovarian tumors.

**Electronic supplementary material:**

The online version of this article (doi:10.1186/s12885-016-2850-8) contains supplementary material, which is available to authorized users.

## Background

Macroautophagy (hereinafter referred to as autophagy) is a highly conserved catabolic process where bulk cellular contents are sequestered in autophagosomes and degraded in autolysosomes [1]. Autophagy has been shown to play and important, but ambiguous, role in tumorigenesis, metastasis, and dormancy. Numerous studies have shown that autophagy is largely inhibitory to tumor growth in the early stages of tumorigenesis, yet promotes established tumor survival during dormancy [2] and in response to pharmacological and metabolic stress [3]. Autophagy is closely linked to other cell death pathways and has been shown to regulate the execution of apoptotic and necroptotic programs [4, 5]. Autophagy may also promote cell death independently of apoptosis and necroptosis (autophagic cell death and autosis) [6, 7].

The tumor suppressor ARHI (DIRAS3) is a potent inducer of autophagy and non-apoptotic cell death in vitro and is down-regulated or lost in over 60 % of primary ovarian tumors [8, 9]. However, assessment of metastatic disease in second look operations has shown that ARHI is up-regulated in over 80 % of these cases [10]. Induction of ARHI in mouse xenograft models of ovarian cancer blocks tumor growth, induces autophagy, and maintains dormancy rather than cell death [2]. Previously, we have shown that ARHI-mediated cell death in culture is dependent on autophagy and the ARHI protein is associated with the RIP1/RIP3 complex [11]. This suggests that ARHI promotes a form of autophagy-associated necroptosis, a phenomenon that has also been described in a pharmacological model of rhabomyosarcoma [12]. In some cell culture models, execution of necroptosis is facilitated by a “metabolic burst” [13] although the precise role of metabolism during ARHI-induced necroptosis is unknown.

While the molecular mechanisms that govern autophagy-dependent cell death during ARHI expression in vitro are beginning to be understood, the role of metabolism in regulating cell death (in vitro) and tumor dormancy (in vivo) remains unclear. Here, we describe the use of nuclear magnetic resonance (NMR), in conjunction with pharmacological inhibition and molecular imaging, to characterize the major metabolic changes resulting from ARHI induction in cell culture and in xenograft models of ovarian cancer. We observe significant up-regulation of glycolysis and glutaminolysis in vitro, both of which are dependent on functional autophagic machinery. In contrast to the metabolic burst associated with other models of necroptotic cell death [13, 14], glycolysis and glutaminolysis appear to support cell survival during ARHI expression in culture. We also observe significant changes in water-soluble choline metabolites in cell culture and tumor models that are the reverse of those typically observed in rapidly proliferating cancer cells and tumors. Increased levels of intracellular free amino acids are observed in cell and tumor lysates following ARHI induction consistent with degradation of cellular proteins during autophagy. To our knowledge, this work represents the first metabolomic analysis of ARHI induction and raises the exciting possibility of targeted metabolic therapy for the detection and suppression of dormant ovarian cancer.

## Results

### Global metabolic changes in SKOv3-ARHI

ARHI expression was induced by doxycycline (Dox) to physiological levels as described previously [2] and resulted in almost 50 % growth inhibition at 48 h (Additional file 1: Figure S1A). In contrast, treatment of parental SKOv3 cells with Dox revealed no significant changes in growth over 48 h indicating that Dox itself has minimal effect on SKOv3 metabolism. These trends are also observed in parental Hey, and Hey-ARHI cells (Additional file 1: Figure S1C). Addition of Dox to SKOv3-ARHI and Hey-ARHI resulted in robust expression of ARHI at 24 h which was accompanied by an increase in LC3 II consistent with induction of autophagy (Additional file 1: Figure S1B, D).

**Fig. 1:**
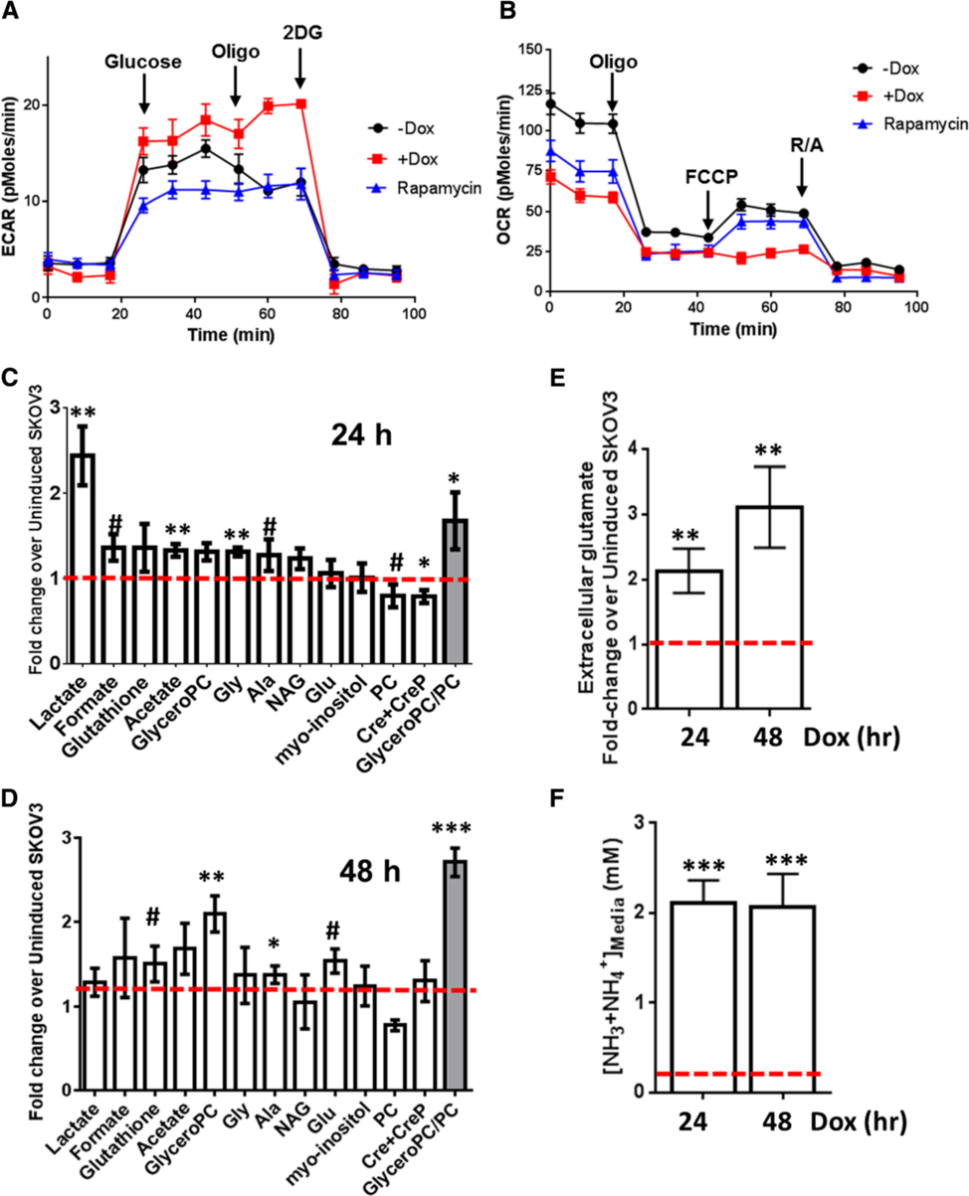
Changes in SKOv3 metabolism after induction of ARHI. SKOv3-ARHI cells pretreated with doxycycline or rapamycin were assayed using Seahorse XF24 analyzer to determine (**a**) ECAR and (**b**) OCR. The relative concentrations of intracellular metabolites at (c) 24 and (**d**) 48 h following ARHI induction were determined by ^1^H-NMR, normalized to viable cell count, and expressed as a fold-change relative to non-induced cells (dotted red line). **e** The concentration of extracellular glutamate in SKOv3-ARHI media at 24 and 48 h post-induction was determined by ^1^H-NMR, normalized to viable cell count, and expressed as a fold-change relative to non-induced SKOv3-ARHI media (dotted red line). **f** The concentration of extracellular ammonia (NH_3_ + NH_4_^+^) in SKOv3-ARHI media at 24 and 48 h post-induction was determined by fluorimetric assay; the ammonia concentration in non-induced media is shown by a dotted red line. The mean values for three replicates (*n* = 3) are shown along with the standard deviation. Statistical significance was determined by unpaired two-tailed *t* test in GraphPad (#, 0.1 > *p* > 0.05; *, *p* < 0.05; **, *p* < 0.01; ***, *p* < 0.001)

SKOv3-ARHI cells in culture showed a higher extracellular acidification rate (ECAR) following Dox induction relative to untreated SKOv3-ARHI controls (Fig. 1a). Treatment of SKOv3-ARHI by rapamycin revealed a lower ECAR relative to untreated cells consistent with a lower rate of glycolysis following mTOR inhibition. Induction of ARHI resulted in a significantly lower oxygen consumption rate (OCR) relative to both untreated cells and cells treated with rapamycin (Fig. 1b). In addition, Dox-treated SKOv3-ARHI cells showed no increase in OCR when treated with the proton gradient uncoupler FCCP. These results suggested that induction of ARHI results in a higher glycolytic rate and lower rate of mitochondrial respiration.

To confirm these initial findings, we carried out a global survey of metabolite levels by ^1^H-NMR to further characterize changes in metabolite levels following induction of ARHI and autophagy in cultured SKOv3-ARHI. ^1^H-NMR of water soluble metabolites 24 and 48 h following ARHI induction in SKOv3 cells revealed significant increases in intracellular lactate and alanine, supporting a transition to a glycolytic phenotype (Fig. 1c and d). We observed a modest increase in formate and acetate, most notably at 24 h. Additionally, we observed an increase in the concentration of glycerophosphocholine (GPC) at 48 h and a concomitant decrease in phosphocholine (PC) (Fig. 1d). Glutathione levels remained consistently elevated at both 24 and 48 h relative to non-induced controls. ^1^H-NMR of the culture media revealed a 3-fold increase in extracellular glutamate at 48 h relative to non-induced SKOv3-ARHI (Fig. 1e). Finally, the concentration of extracellular ammonia increased by approximately 10-fold over non-induced controls (~2 mM vs. 0.2 mM) at 24 and 48 h (Fig. 1f).

### ARHI induction results in up-regulation of glycolysis

**Fig. 2:**
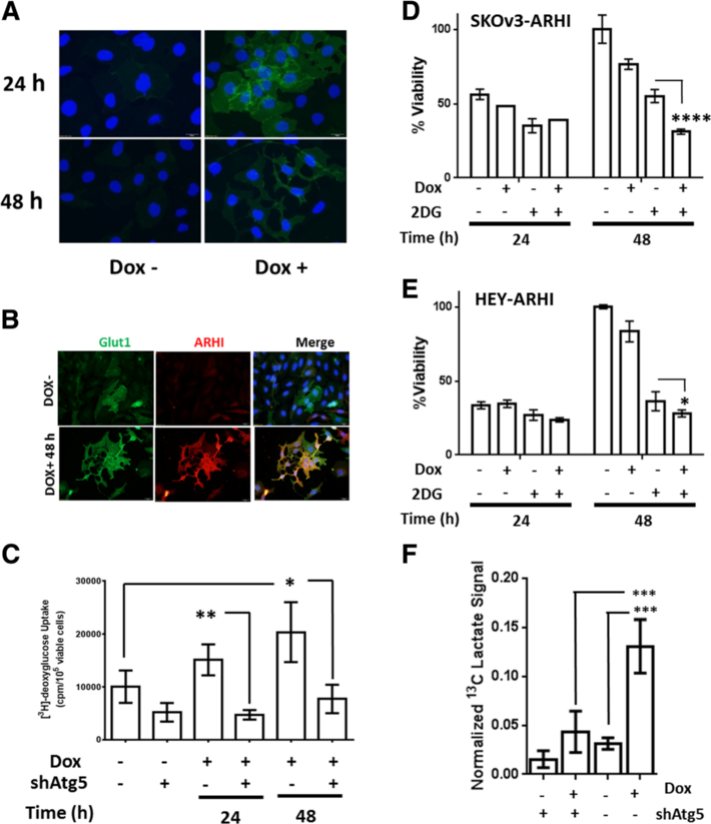
ARHI induction increases glucose uptake and glycolytic activity. **a** Immunofluorescent staining of SKOv3-ARHI cells for GLUT1 (*green*) at 24 and 48 h post-induction with doxycycline (DOX). The nuclei are stained with DAPI (*blue*) for reference. **b** Immunofluorescent staining of SKOv3-ARHI cells for GLUT1 and ARHI at 48 h post-induction. **c** SKOv3-ARHI cells were induced with Dox at the given time points and the uptake of [^3^H]-2-deoxyglucose determined. SKOv3-ARHI cells were stably transfected with shRNA for Atg5 to block the initiation of autophagy (shATG5). Uptake was measured as cpm/10^5^ viable cells after 30 min of incubation. Three independent samples for each condition were used (*n* = 3) to compute the mean and standard deviation and the statistical difference in uptake between samples was determined by unpaired two-tailed *t* test (*, *p* < 0.05. **, *p* < 0.01). **d** SKOv3-ARHI or **e** HEY-ARHI cells were treated with 2-deoxglucose (2-DG) and induced with Dox in media for the specified times (*n* = 3 for each condition). Percent cell viability was determined by SRB assay. Statistical difference was determined by unpaired two-tailed *t* test (*, *p* < 0.05; ****, *p* < 0.0001). **f** Normalized extracellular lactate signal measured by ^13^C-NMR between 24 and 32 h of ARHI induction autophagy. Three independent samples were for each condition were used to calculate the mean and standard deviation and statistical significance was determined by two-way ANOVA with p-values adjusted for multiple comparisons (***, *p* < 0.001)

Based on the results of our preliminary metabolomic survey, we investigated the changes in glucose metabolism in the SKOv3 model of ARHI expression. Immunofluorescent microscopy detected a significant increase in staining of the GLUT1 high-affinity glucose transporter in ARHI-induced SKOv3 cells beginning 24 h following ARHI induction (Fig. 2a). In a separate experiment, cells were co-stained for GLUT1 and ARHI expression after 48 h to confirm that altered GLUT1 staining was not the result of clonal selection (Fig. 2b). Western blot analysis showed only a modest increase in total cellular GLUT1 (Additional file 1: Figure S2) suggesting that increased GLUT1 staining may be the result of altered receptor localization or availability on the cell surface. To determine if altered GLUT1 expression was correlated with increased glucose uptake, we measured the uptake of the radiolabeled glucose analog [^3^H]-2-deoxyglucose. As seen in Fig. 2c, [^3^H]-2-deoxyglucose uptake increased progressively after ARHI induction. To determine if changes in glucose uptake were the result of ARHI-mediated autophagy (rather than a pleiotropic effect of ARHI), we also included SKOv3-ARHI cells stably transfected with shRNA against the Atg5 protein. Suppression of Atg5 expression dramatically reduced autophagy initiation (Additional file 1: Figure S3) and knockdown of Atg5 in this experiment resulted in almost complete reversal of enhanced glucose uptake. These observations also suggested that the inhibitory effects of non-metabolizable glucose analogs such as 2-deoxyglucose (2-DG) would be highest in autophagic SKOv3-ARHI cells at 48 h post-induction, where glucose uptake is maximal. SKOv3-ARHI cells were treated with 10 mM 2-DG along with Dox to determine the effect of ARHI expression on sensitivity to glycolysis inhibition. As seen in Fig. 2d, a combined inhibitory effect of Dox and 2-DG is observed at 48 h. This effect is also observed in Hey-ARHI cells at 48 h, although sensitivity to the combination of 2-DG and ARHI expression was less dramatic (Fig. 2e) possibly due to the lower expression of ARHI in this line relative to SKOv3-ARHI (Additional file 1: Figure S1D). In a separate set of experiments we sought to determine the relationship between ARHI expression, autophagy, and lactate production. In these experiments, SKOv3-ARHI cells stably transfected with either control (scrambled) shRNA or shRNA specific for the Atg5 protein were treated with or without Dox. Accumulation of lactate in the media between 24 and 32 h was measured ^13^C NMR. As seen in Fig. 2f, ARHI expression resulted in a 4-fold increase of extracellular lactate which was almost completely reversed in the stable Atg5 knockdown cells. Western blot analysis showed only modestly increased expression of lactate dehydrogenase (LDH) (~ 25 %) at 24 h relative to non-induced controls (Additional file 1: Figure S4).

**Fig. 3:**
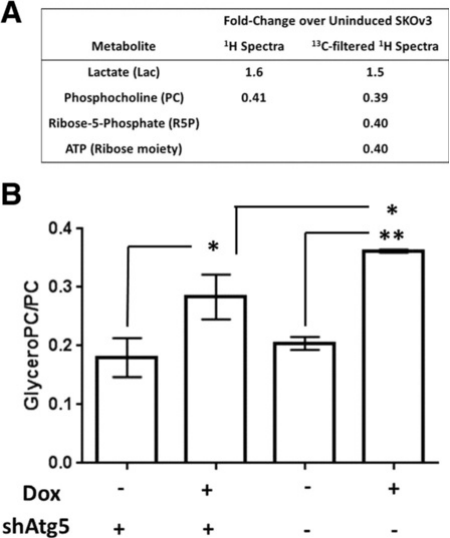
ARHI induction alters glucose and choline metabolism. **a** SKOv3-ARHI cells were induced with Dox or uninduced for 48 h followed by the addition of ^13^C_6_-glucose in the media. The intracellular water-soluble metabolite concentrations were determined by ^1^H and ^13^C-filtered ^1^H NMR spectra (1D HSQC) after 8 h, normalized to cell count, and expressed as a ratio between induced and uninduced samples. **b** Glycerophosphocholine:phosphocholine (GPC/PC) ratio in water-soluble extracts of SKOv3-ARHI cells transfected with shAtg5 or a control shRNA. Data was obtained by ^1^H NMR of water soluble metabolite extracts of SKOv3-ARHI between 24 and 48 h in culture. Three replicate samples were used for each condition to calculate the mean and standard deviation and statistical significance was determined by two-way ANOVA with p-values adjusted for multiple comparisons (*, *p* < 0.05; **, *p* < 0.01)

Our observation of increased glucose uptake and lactate production suggested that glycolysis is up-regulated during ARHI-induced autophagy. To confirm this, we performed feeding studies on SKOv3-ARHI cells with universally labeled ^13^C_6_-glucose in conjunction with ^13^C-filtered ^1^H spectra (1D-HSQC) analysis of the resulting water-soluble metabolites. In these experiments, the ^1^H-proton spectra is effectively edited to show only signal from protons directly bonded to ^13^C carbon atoms. Briefly, cells were treated with Dox for 48 h to induce ARHI, at which time the medium was replaced with glucose-free media supplemented with 20 mM ^13^C_6_ D-glucose. After 8 h the cells were harvested and the water-soluble metabolites analyzed by 1D-HSQC. As seen in Fig. 3a, incorporation of the ^13^C label into lactate was significantly enhanced in Dox-treated SKOv3 cells confirming our previous results (Fig. 2f). ^13^C_6_ D-glucose feeding experiments also showed approximately 60 % reduction in the labeling of ribose-5-phosphate and the ribose moiety of ATP in Dox-treated cells suggesting a reduction of glucose flux through the pentose-phosphate pathway (PPP). These experiments also showed >60 % reduction phosphocholine in Dox-treated SKOv3 cells relative to controls although this signal resulted from natural abundance ^13^C and not from the labeled glucose.

Given the significant changes in phosphocholine observed in the 1D HSQC experiment, we sought to determine the changes in the ratio of intracellular glycerophosphocholine to phosphocholine (GPC/PC). As seen in Fig. 1c and d, this ratio increases within 24 h of ARHI induction relative to the non-induced control cells and is over 2.5-fold greater than the non-induced controls after 48 h as measured by ^1^H-NMR. We determined the GPC/PC ratio in SKOv3-ARHI shAtg5 knockdown cells with and without Dox induction for 48 h. As seen in Fig. 3b, induction of ARHI resulted in an approximately 2-fold increase in GPC/PC relative to non-induced controls which was consistent with our initial measurements. This effect was diminished by Atg5 knockdown although a significant increase in GPC/PC is still observed during autophagy inhibition. Western blot analysis showed a modest reduction (~10–15 %) in choline kinase α (CK) expression during the same time period following ARHI induction (Additional file 1: Figure S4).

### ARHI induction enhances glutamine uptake and glutamate production

Our initial ^1^H-NMR survey experiments indicated a substantial increase extracellular glutamate during ARHI induction (Fig. 1e) suggesting up-regulation of glutaminolysis. To further explore changes in glutamine metabolism, SKOv3-ARHI cells were induced with Dox for 24 h followed by growth in glutamine-free media supplemented with 2 mM 5-^13^C-labeled glutamine for an additional 24 h under Dox induction. ^13^C-NMR of soluble metabolites and culture media after 24 h of incubation with labeled 5-^13^C-glutamine were employed to determine the uptake and metabolic fate of the labeled carbon.

**Fig. 4:**
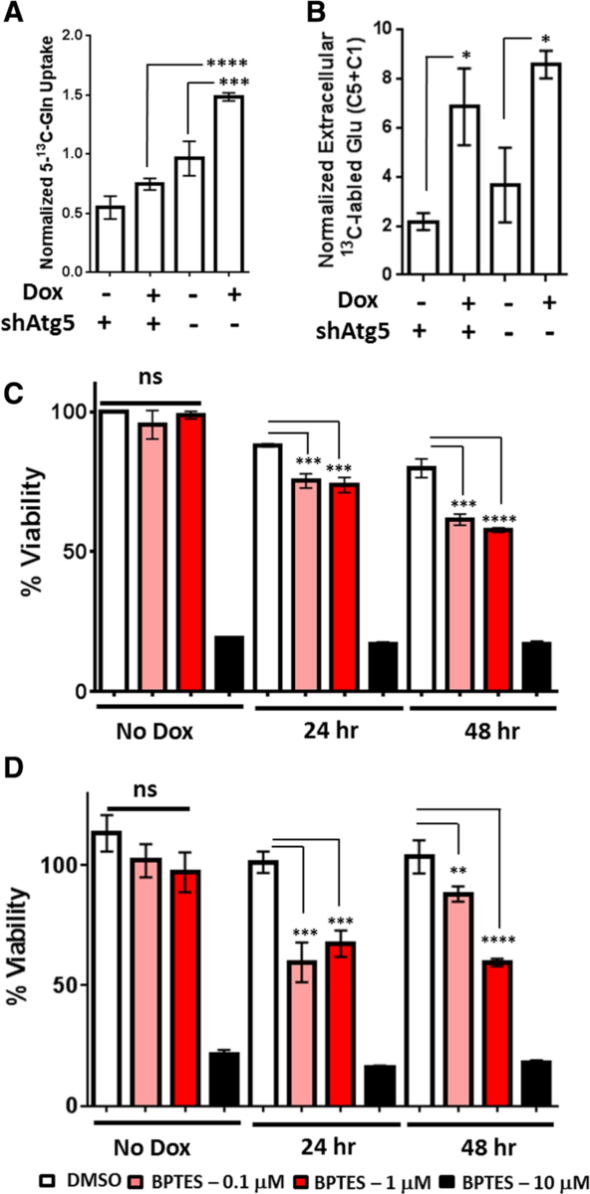
ARHI Expression Increases Glutamine Uptake and Glutamate Excretion in SKOv3-ARHI. **a** Quantitation of 5-^13^C-glutamine uptake in SKOv3-ARHI between 24 and 48 h in culture by ^13^C NMR. Uptake was calculated by subtracting the media Gln C5 carbon-13 signal at 48 h from the media signal at 24 h and normalized to the total media proton signal. **b** Normalized concentration of ^13^C-labeled extracellular glutamate derived from 5-^13^C-glutamine after incubation with 5-^13^C-glutamine for 24 h. Values were obtained by adding the integrated C1 and C5 glutamate resonances and dividing by the total proton signal in the media. Three replicates were used for each condition to calculate the mean and standard deviation. **c** Effect of Glutaminase inhibition on SKOv3-ARHI and **d** HEY-ARHI cell viability. BPTES was incubated with cells at the concentrations shown in the presence and absence of Dox induction for 24 and 48 h. Cell viability was determined by MTT assay. Each experimental condition was carried out in triplicate and used to calculate the mean and standard deviation. Statistical significance was determined by two-way ANOVA and p-values adjusted for multiple comparisons (*, *p* < 0.05; **, *p* < 0.01; ***, *p* < 0.001; ****, *p* < 0.0001)

**Fig. 5:**
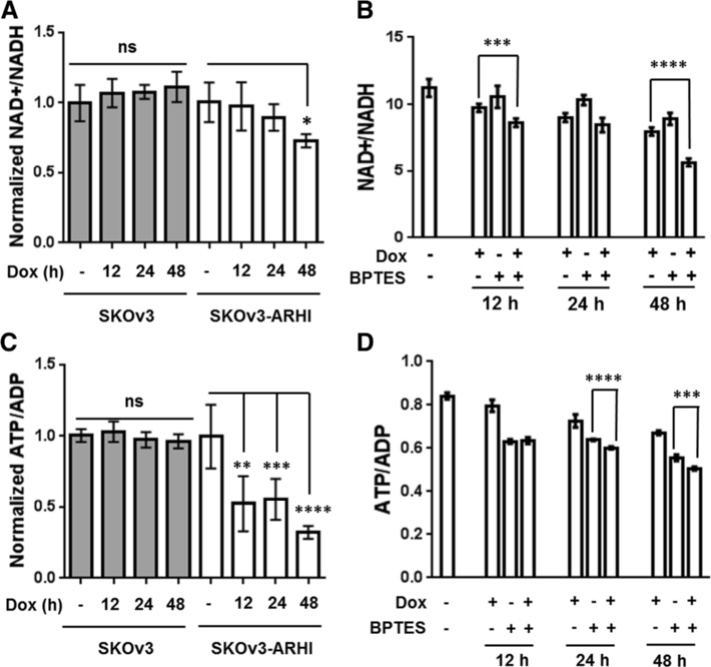
Effect of ARHI Induction and Glutaminase Inhibition on Cellular Energetics. The intracellular NAD+/NADH **a** and ATP/ADP **c** was determined in for both SKOv3 (parental) and SKOv3-ARHI cells at 12, 24, and 48 h following treatment with Dox (See Methods for additional details). Both ratios were normalized to the values obtained in the Dox- control samples. The same measurements were made for SKOv3-ARHI cells following treatment with Dox, BPTES (1 μM), or both in combination for 48 h (**b** and **d**). Seven replicate samples were used for each condition to calculate the mean and standard deviation and statistical significance was determined by two-tailed *t*-test in GraphPad (*, *p* < 0.05; **, *p* < 0.01; ***, *p* < 0.001; ****, *p* < 0.0001)

As seen in Fig. 4a, uptake of 5-^13^C-glutamine was enhanced approximately 1.5-fold relative to non-induced control SKOv3-ARHI in the 24─48 h period following ARHI induction. Atg5 knockdown resulted in a 2-fold reduction in labeled glutamine uptake indicating a signficant role of autophagy in mediating enhanced glutamine uptake. Labeled extracellular glutamate was likewise increased >2-fold in ARHI-expressing cells during the same period (Fig. 4b), although Atg5 knockdown failed to produce a statistically signficant reduction in this effect. Treatment of non-induced SKOv3-ARHI and Hey-ARHI cells with the glutaminase inhibitor BPTES in the absence of autophagy resulted in a substantial decrease in cell viability at 10 μM but no effect at either 1 μM or 0.1 μM (Fig. 4c and d). After ARHI induction, both cell lines showed signficantly reduced viability in the presence of 1 μM and 0.1 μM BPTES at 24 and 48 h post-induction with almost 50 % reduction in viability seen at the 48 h time point. To determine the effect of BPTES inhibtion on cellular bioenergetics, we determined the intracellular NAD+/NADH and ATP/ADP ratios in SKOv3-ARHI cells treated with Dox, BPTES, or a combination of both (Fig. 5b and d). Induction of ARHI by Dox resulted in a time-dependent decrease in the NAD+/NADH ratio which was significantly enhanced by the addtion of BPTES particularly after 48 h (Fig. 5b). A similar trend in the ATP/ADP ratio was observed although the additive effect of ARHI induction and BPTES was more modest (Fig. 5d). Taken together, the decline in both ratios suggests a progressive inhibtion of cellular metabolism following ARHI induction which is exacerbated by inhibtion of glutamine metabolism.

### ARHI expression modulates mitochondrial membrane potential and intracellular oxidative state

**Fig. 6:**
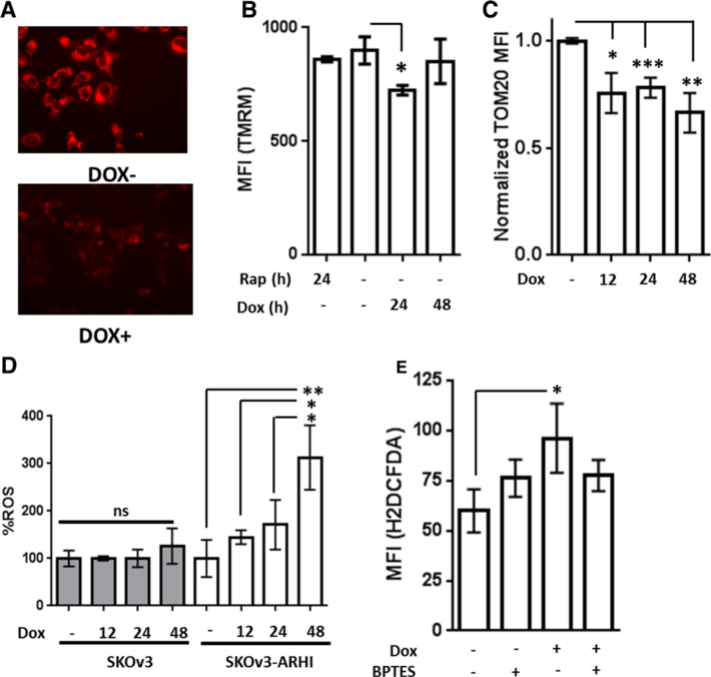
ARHI Expression alters Mitochondrial Membrane Potential and ROS in SKOv3-ARHI. **a** SKOv3-ARHI cells were treated with and without Dox followed by staining with Mitotracker Red FM and visualized by fluorescence microscopy at 24 h. **b** SKOv3-ARHI cells were treated with Dox or Rapamcyin for the indicated times followed by tetramethyl rhodamine methyl ester (TMRM). The mean fluorescence intensity (MFI) was obtained by flow cytometry. Three replicate samples were used for each condition and the error bars represent the standard deviation. Statistical significance was obtained by two-tailed *t*-test in GraphPad (*, *p* < 0.05) **c** SKOv3-ARHI cells were treated with Dox for 48 h, followed by fixation in 3 % paraformaldehyde and permeabilization with 0.1 % Triton X-100. Cells were then stained with TOM20 antibody (Santa Cruz Biotechnology) and a secondary goat Anti-rabbit IgG conjugated to AF488® and analyzed by flow cytometry. Eight replicates from two independent experiments were normalized to the MFI obtained in the Dox- controls and analyzed in GraphPad. The error bars represent the standard error and statistical significance was obtained by a two-tailed *t*-test (*, *p* < 0.05; **, *p* < 0.01; ***, *p* < 0.001). **d** SKOv3 (parental) and SKOv3-ARHI cells were treated with Dox for the indicated times followed by H2DCFDA and analyzed by flow cytometry. The resulting MFI values were normalized to the Dox- value in either the parental or ARHI-transfected cell line. **e** SKOv3-ARHI cells were treated with Dox for 48 h with and without BPTES (1 uM) followed by staining with H2DCFDA and analysis by flow cytometry. Each experimental condition in **d**) and **e**) was carried out in triplicate and the mean fluorescence intensity plotted above along with the standard deviation. The statistical significance was determined by unpaired, two-tailed *t*-test in GraphPad (*, *p* < 0.05; **, *p* < 0.01)

Staining of SKOv3-ARHI cells with Mitotracker dye following induction of ARHI revealed a signficant decrease in dye uptake suggesting a loss of mitochondrial membrane potential (Fig. 6a). To quantitate this effect, SKOv3-ARHI cells were treated with Dox followed by the nernstian fluorescent probe TMRM and analyzed by flow cytomtery. As seen in Fig. 6b, treatment with Dox resulted in a decrease in TMRM uptake which was most pronounced at 24 h post-induction. In constrast, treatement with rapamycin had no effect on TMRM uptake. In order to determine if low TMRM uptake was being driven by low mitochondiral membrane potential or by a decrease in the number of mitochondria, SKOv3-ARHI cells were fixed and permeabilized following induction with Dox for 12, 24, and 48 h. The cells were then stained for the presence of the TOM20 receptor of the mitochondrial outer membrane preprotein translocase and analyzed by flow cytometry. As seen in Fig. 6c, significantly lower Tom20 signal was observed after 12 h of Dox induction suggesting a decrease in the mitochondrial mass following ARHI induction. We also sought to detemine the effect of ARHI induction on the intracellular oxidative state using the oxygen-sensitive dye H2DCFDA. Induction of ARHI was found to increase H2DCFDA uptake by flow cytometry by almost 3-fold after 48 h, in good agreement with our previous work (Fig. 6d) [11]. In contrast, parental SKOv3 cells showed no statisitically significant change in H2DCFDA following treatement with Dox indiciating that changes in intracellular redox state were the result of ARHI expression. Finally, we measured H2DCFDA uptake following ARHI induction and BPTES treatment for 48 h (Fig. 6e). We found that BPTES treatment resulted in decreased H2DCFDA uptake in Dox-induced SKOv3 cells while BPTES treatment alone resulted in no statistically significant change.

### Metabolic effects of ARHI expression in In vivo xenograft models of ovarian cancer

**Fig. 7:**
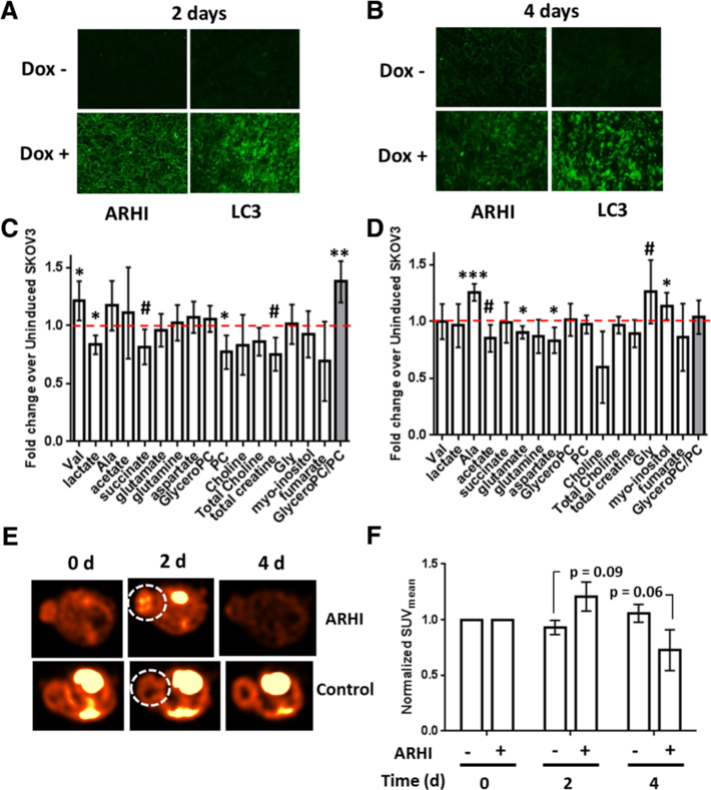
In vivo Metabolomics of SKOv3-ARHI. Tumors obtained from mice treated with doxycycline and sucrose (Dox+) or sucrose alone (Dox-) for 2 days (**a**) or 4 days (**b**) were sectioned and stained with fluorescent anti-ARHI and anti-LC3 antibodies (green). Tumors at 2 days (**c**) or 4 days (**d**) following treatment were homogenized and extracted to yield the water-soluble metabolites which were analyzed by ^1^H-NMR. After normalizing each metabolite to the total ^1^H-NMR signal, the fold-change ratio was calculated to determine the relative change in each metabolite after ARHI induction relative to non-induced tumors (*dotted red line*). The mean fold-change between induced (*n* = 4) and non-induced (*n* = 4) tumors at each time point is plotted along with the standard deviation. Statistical significance was determined by unpaired two-tailed *t*-test in Graphpad (#, 0.1 > *p* > 0.05; *, *p* < 0.05; ***, *p* < 0.001). **e** Subcutaneous SKOv3-ARHI were injected with [^18^F]-FDG and imaged by PET/CT at 0, 2, and 4 days following induction of ARHI. Axial slices from a representative ARHI-induced (*n* = 4) or control (*n* = 5) mouse in each group are shown for each of the time points. The tumor is circled (*white dotted line*). **f** The mean standard uptake value (SUV_mean_) in each tumor was determined as a function of time and normalized to the SUV_mean_ at time = 0. The normalized values were plotted along with the standard error of the mean. Statistical significance between induced and control groups at 2 days and 4 days was determined by two-way ANOVA with p-values adjusted for multiple comparisons

We employed the SKOv3-ARHI xenograft mouse model to measure the changes in intracellular metabolites following induction of ARHI in vivo. Based on a time course of ARHI expression in vivo, we chose to begin our metabolic analyses at 48 h where ARHI expression is signficantly above background (Additional file 1: Figure S5). Subcutaneous SKOv3-ARHI mice were given Dox in drinking water and groups of four were sacrificed at 2 days and 4 days post-induction. The tumors were removed and the water-soluble metabolites extracted and analyzed by ^1^H-NMR. Tumors from the Dox-treated group showed robust expression of ARHI and LC3 throughout the tumor as evdienced by immunohistochemical fluorescence staining. Tumors from the control group showed almost no ARHI signal and low LC3 signal as expected (Fig. 7a, b). ^1^H-NMR analysis of the water-soluble metabolites from ARHI-induced tumors at 2 days (Fig. 7c) showed signficant increases in the concentration of valine and alanine relative to the non-induced controls. Lactate, succinate, phosphocholine, and total creatine all showed significant decreases. At 4 days post-induction (Fig. 7d) alanine, glycine, and myo-inositol were significantly increased while acetate, glutamate, aspartate, and choline were decreased. We observed an approximately 1.5-fold increase in the glycerophosphocholine:phsophocholine ratio two days post-induction which disappeared by day four.

### [^18^F]-FDG PET/CT Imaging

To determine the effect of ARHI expression on glucose uptake in vivo, we employed positron emission tomography/computed tomography (PET/CT) using the non-metabolizable probe [^18^F]-FDG. Following a baseline scan, mice were divided into control (*n* = 5) and ARHI-induced (*n* = 4) groups and rescanned at 2 and 4 days. At 2 days, the ARHI-induced group showed a ~20 % increase in [^18^F]-FDG uptake in the tumor (Fig. 7e) which decreased below the initial baseline level by 4 days. The control tumors showed minimal changes in FDG uptake over the 4 day period. Figure 7f shows the normalized mean Standard Uptake Values (SUV_mean_) for each group at the three time points.

## Discussion

In this work, we observe broad-based metabolic changes over the course of 48 h following induction of ARHI expression in cell culture. After 48 h, we observe a significant reduction in cell viability which limited our ability to accurately measure metabolite levels beyond this time point. In the case of subcutaneous SKOv3-ARHI tumors, ARHI induction had minimal effect on tumor viability allowing us to measure metabolite levels even after 4 days. This paradoxical observation underscores the fundamental difference between the biological effects of ARHI expression and autophagy in vitro (cell death) and in vivo (dormancy).

### In vitro metabolomics of ARHI-induced autophagy – glycolysis

Our previous work demonstrated that ARHI-mediated necroptotic cell death in vitro requires functional autophagic machinery as well as the RIP1/RIP3 complex [11]. In some cell culture models, RIP1/RIP3-mediated necroptotic cell death has been shown to be accompanied by a “metabolic burst” characterized by enhanced glycolysis, glutaminolysis, and ROS production which supports this cell death program [15]. In this work, we observe that ARHI expression results in increased glucose uptake and lactate production and that inhibition of autophagy by Atg5 knockdown reversed both effects (Figs. 1 and 2). This suggests that autophagy is playing a key role in governing glucose metabolism during ARHI induction. Increased glucose uptake was not correlated to increased flux through the pentose phosphate pathway (PPP); indeed we observed diminished incorporation of the ^13^C label from ^13^C_6_-glucose into ribose-5-phosphate by 1D-HSQC ^1^H-NMR (Fig. 3a). However, rather than attenuating cell death, glycolysis inhibition by 2-DG resulted in a further reduction in cell viability (Fig. 2d, e). Based on this data, we conclude that ARHI expression and autophagy contribute to increased glycolytic metabolism which may support cell viability.

It has been previously observed that induction of autophagy in apoptosis-deficient cells following growth factor withdrawal results in down-regulation of glucose uptake and glycolysis [16]. However, other recent reports have shown that autophagy supports glycolysis in Ras-transformed cells [17] and in muscle cells during exercise [18]. It has also been shown that glycolysis can be up-regulated during autophagy to maintain intracellular ATP in response to loss of mitochondrial membrane potential (∆Ψ) and respiratory function [19]. We observe that ARHI induction results in a substantially lower OCR and higher ECAR in SKOv3 cells (Fig. 1a, b) which is accompanied by a reduction in ATP/ADP and NAD+/NADH (Fig. 5a, b). This loss of mitochondrial respiration and subsequent increase in energetic demand would be expected to result in activation of AMPK, which itself is a known activator of glycolysis and GLUT1 expression [20]. Indeed, we observe AMPK phosphorylation following ARHI expression [2] as well as increased phosphorylation of the AMPK substrate acetyl CoA carboxylase (ACC) (Additional file 1: Figure S6).

While it is difficult to draw firm mechanistic conclusions from the data in this work, our working hypothesis is that ARHI expression triggers a bioenergetic shock by direct inhibition of mitochondrial function or by initiating mitochondrial depletion through autophagy. In support of direct mitochondrial inhibition, it has been previously shown that ARHI expression results in dephosphorylation and nuclear translocation of FOXO3a [21] which down-regulates the expression of mitochondrial proteins and respiratory complex activity [22]. FOXO3a activity also results in transient mitochondrial membrane permeabilization [23] and ROS formation [24] which we observe in response to ARHI expression (Fig. 6b and d). Alternatively, ARHI expression has also been shown to alter the subcellular localization of STAT3 [25] which plays an important role in maintaining the activity of Complex I and II in the electron transport chain [26]. Either of these mechanisms could explain the observed bioenergetic shock although additional experiments are necessary to determine which (if any) of these are relevant in the ARHI model.

Depletion of functional mitochondria by excessive autophagy represents a second mechanism by which a bioenergetic shock could occur. Indeed, removal of mitochondria by autophagy in cellular models of senescence [27] and during erythrocyte maturation [28] has been shown to trigger a glycolytic phenotype. This phenomenon has also been observed in cell models of K-Ras transformation where autophagic depletion of mitochondria results in increased glycolysis and decreased oxygen consumption which was rescued by pharmacological inhibition of autophagy [29]. In this work, we observe a reduction in TOM20 staining following ARHI induction (Fig. 6c) as well as a decreased TMRM uptake and Mitotracker staining (Fig. 6a, b) indicating a drop in mitochondrial mass, depolarization of the mitochondrial membrane potential, or a combination of the two. The autophagy-dependence of glycolysis up-regulation during ARHI induction also supports this model although additional experiments are necessary to clarify the effects of ARHI-mediated autophagy on mitochondrial mass and bioenergetics.

### In vitro metabolomics – glutaminolysis

RIP1/RIP3-dependent necroptosis has also been shown to activate glutamate dehydrogenase 1 (GLUD1) [14], leading to the hypothesis that increased glutaminolysis is a key component of the metabolic burst that facilitates necroptotic cell death [13, 15]. In this work, we have shown that expression of ARHI significantly increases the uptake of glutamine from the media as well as the concentration of extracellular glutamate (Figs. 1e and 4a, b). In contrast to the RIP1/RIP3 metabolic burst hypothesis, ARHI expression sensitizes both autophagic SKOv3-ARHI and Hey-ARHI cells to growth inhibition by glutaminolysis blockade (Fig. 4c, d). strikingly, we observed nearly 50 % reduction in cell viability with 1 μM BPTES in autophagic cells while non-autophagic cells show no response at this concentration. BPTES treatment alone results in a modest decrease in intracellular NAD+/NADH at 48 h post-treatment and a more significant, but time-independent, decrease in ATP/ADP. Concomitant Induction of ARHI results in a further decrease in both ratios suggesting that glutaminase inhibition is amplifying the metabolic stress resulting from expression of ARHI. This further suggests that up-regulation of glutamine metabolism, rather than promoting the necroptotic cell death program, serves a pro-survival function by providing a source of TCA metabolites through anaplerosis [30].

The TCA cycle interemediates oxaloacetate and α-ketoglutarate can also be used as precursors for the synthesis of amino acids (cataplerosis) [31]. 5-^13^C-glutamine feeding studies showed significantly reduced ^13^C label incorporation into intracellular aspartic acid (C4 and C1) and glutamate (C1) following ARHI induction which was almost completely reversed by Atg5 knockdown (Additional file 1: Figure S7). This may indicate reduced cataplerotic biosynthesis of amino acids during ARHI-mediated autophagy. Alternatively, this effect may result from a dilution of the ^13^C label in newly synthesized amino acids by increased TCA entry of non-labeled amino acids derived from autophagic degradation of intracellular proteins. This is supported by the large increase in extracellular ammonia following ARHI induction (Fig. 1f) which could arise from increased deamination of free amino acids [32] as well as increased glutaminolysis. While additional feeding experiments are necessary to full characterize TCA cycle flux, our data indicates decreased cataplerotic biosynthesis of amino acids following induction of ARHI and autophagy.

Glutaminolysis can also serve as an adaptive response to oxidative stress by providing reducing power in the form of NADPH through oxidation of malate to pyruvate [30, 33]. This glutamine-dependent pathway has recently shown to support the growth of KRAS-transformed pancreatic cancer cells by increasing the ratio of NADPH/NADP+ which attenuates ROS toxicity though maintenance of reduced glutathione [33]. While we did not directly observe malate in our NMR experiments, we consistently observed increased intracellular glutathione levels (Fig. 1c, d) as well as increased incorporation of 5-^13^C-glutamine-derived label into intracellular glutathione following ARHI induction (Additional file 1: Figure S7). However, when SKOv3-ARHI cells were treated with both Dox and BPTES, H2DCFA uptake was almost completely restored to basal levels (Fig. 6e) indicating that increased glutamine flux is primarily directed to the TCA cycle. Taken together, our data show that ARHI-mediated autophagy results in up-regulation of glutaminolysis and decreased cataplerosis suggesting an adaptive response to increased energetic demand.

### In vitro metabolomics – choline metabolism

Proliferating ovarian cancer cells, like many other transformed cells, show increased phosphocholine production, likely due to increases in choline kinase (CK) activity [34]. A rise in phosphocholine is correlated with increased rates of phosphatidylcholine synthesis and lipid membrane biogenesis to support increased rates of cell growth and division. In contrast, we observe significantly decreased phosphocholine and increased glycerophosphocholine levels (Figs. 1c, d and 3b), indicating a switch from lipid biosynthesis to lipid catabolism. This is supported by the decreased levels and increased phosphorylation (inactivation) of Acetyl-CoA Carboxylase (ACC) during ARHI expression (Additional file 1: Figure S6). Decreased PC may also arise from inhibition of the PI3K pathway by ARHI [35, 36], although Atg5 knockdown data (Fig. 3b) suggests that autophagy is also playing a role in altering lipid metabolism. Alterations in choline metabolism, represented by an increased glycerophosphocholine:phosphocholine (GPC:PC) ratio, suggest that expression of ARHI results in a shift away from a proliferative metabolic phenotype which may have implications for ARHI-driven tumor dormancy.

### In vivo metabolomics of ARHI-induced autophagy

We next sought to determine the correlation between ARHI-mediated metabolic changes in vitro and in vivo. ARHI expression was readily induced by Dox in SKOv3-ARHI subcutaneous tumors within two days and was positively correlated with expression of LC3 (Fig. 7a, b). Analysis of the soluble metabolites two and four days post-induction revealed modest but statistically significant increases in the concentrations of free valine, glycine, and alanine in autophagic SKOv3 tumors (Fig. 7c, d) consistent with cell culture studies (Fig. 1c, d). These likely arise from bulk degradation of proteins in the autophagosome and the subsequent export of di- and tri-peptides into the cytosol for processing into amino acids for catabolic and anabolic use. We also observed a ~1.5 fold increase in the GPC:PC ratio at two-days post-induction consistent with our in vitro data. This returned to baseline within four days post-induction, driven mainly by an increase in the levels of phosphocholine rather than a decrease in glycerophosphocholine. This was in contrast to the pattern observed in vitro, where the GPC:PC ratio was driven by both metabolites and increased over time.

In contrast to SKOv3-ARHI cells cultured in vitro*,* we did not observe a significant increase in the levels of glutamate or lactate in autophagic tumors in vivo (Fig. 7c, d). This may indicate reduced glycolytic and glutaminolytic activity in xenograft tumors relative to cell culture models although we cannot eliminate the possibility of extracellular metabolite washout. Imaging of SKOv3-ARHI tumors by FDG-PET did reveal an increase in [^18^F]-FDG uptake in ARHI-expressing tumors after 2 days although this did not reach the level of statistical significance (*p* = 0.09). After 4 days, [^18^F]-FDG uptake in ARHI-expressing tumors was lower while uptake in the control group was slightly higher which suggests a minor and transient increase in glucose uptake in vivo followed by down-regulation. While these results are broadly consistent with our in vitro observations, the magnitude of change is somewhat lower. Decreased nutrient availability in the tumor microenvironment, in contrast to cell culture conditions, may play a role in governing the metabolic switch to glucose and glutamine utilization in ARHI-expressing tumors. Efficient detoxification of ROS, lactate, and ammonia, perhaps by export to the tumor microenvironment [37], may also affect metabolic reprogramming in vivo. Additional metabolomic experiments, in conjunction with autophagy inhibition, will be necessary to conclusively assess the status of both pathways in tumor models.

## Conclusion

ARHI has previously been shown to mediate autophagy-associated necroptosis in vitro and tumor dormancy with autophagy in vivo. In this work, we present the first metabolic analysis of ARHI expression which revealed evidence of significant bioenergetic perturbation, oxidative stress, and mitochondrial dysfunction in cell culture. ARHI-mediated up-regulation of glycolysis and glutaminolysis was found to be autophagy-dependent and inhibition of these metabolic pathways resulted in decreased cell viability. This may indicate that metabolic rewiring, rather than supporting the necroptotic death program, is an adaptive response to ARHI- and/or autophagy-associated bioenergetic shock and that the commitment to necroptotic cell death is tied to the inability of this response to restore cellular bioenergetics.

Many metabolic changes (e.g. choline metabolism) were found to exhibit a marked time-dependence in both cell culture and animal models suggesting that metabolic rewiring during ARHI expression is a dynamic process. Others (*e.g.* glycolysis and glutaminolysis) undergo significant up-regulation in vitro but show modest or no enhancement in vivo illustrating the importance of biological context in the study of autophagy. In addition to providing a foundation for future experiments to determine the mechanism of ARHI-mediated metabolic perturbation, these results suggest that targeted inhibition of metabolic pathways such as glutaminolysis, may be a powerful approach to overcoming ARHI-mediated tumor dormancy.

## Methods

### Chemicals

Chemicals were used without further purification form commercial vendors: 5-^13^C-glutamine (Cambridge Isotopes), U-^13^C_6_-glucose (Cambridge Isotopes), Doxycycline (Dox, Sigma), D_2_O (Sigma) and DSS-d_6_ (3-(trimethylsilyl)-1-propanesulfonic acid-d_6_ disodium salt, Sigma).

### Cell cultures

Tet-on inducible SKOv3-ARHI ovarian cancer cells were grown in McCoy’s medium supplemented with 10 % FBS, 200 μg/mL G418 (Geneticin) and 0.12 μg/mL puromycin (SKOv3-ARHI medium). Tet-on inducible Hey-ARHI ovarian cancer cells were cultured in RPMI-1640 medium supplemented with 10 % FBS, 25 μg/mL blasticidin and 1 μg/mL puromycin (Hey-ARHI medium). Stable ATG5 knockdown and non-targeted control cell lines were generated by transducing SKOv3-ARHI or Hey-ARHI ovarian cancer cells with lentivrius encoding each shRNA (shATG5 Fisher #V3LHS_301131; shControl Fisher # RHS4348). Cells were propagated in medium, and GFP positive cells were sorted by flow cytometry prior to the experiments. ATG5 protein levels were measured by Western immunoblotting to assess the degree of knockdown in each experiment (see Additional file 1: Figure S3).

### Seahorse measurements

2 × 10^4^ SKOv3-ARHI cells were seeded onto a 24-well XF assay plate and were allowed to attach firmly to the plate for two days before any treatment. 1 μg/mL Doxycycline was added 48 h before Seahorse analysis to induce ARHI expression. 50 nM Rapamycin was added 24 h before analysis to induce autophagy by inhibiting mTOR. XF24 sensor cartridges were hydrated in 1 mL of XF Calibrant at 37 °C without CO_2_ overnight. XF DMEM assay medium was warmed to 37 °C and adjusted pH to 7.4. Cells were washed and equilibrated in 525 μL of assay medium at 37 °C without CO_2_ one hour before analysis. Reagents for the glycolysis and mitochondrial function tests were prepared according to manufacturer’s instruction. Media and reagents were prepared freshly on the day of analysis. To measure cellular glycolytic activity, 75 μL of 80 mM glucose, 18 μM oligomycin and 500 mM 2-deoxy-glucose were injected into assay media sequentially to achieve final concentration of 10 mM, 2 μM and 50 mM, respectively. To determine mitochondrial function, 75 μL of 16 μM oligomycin, 18 μM carbonyl cyanide-p-trifluoromethoxyphenylhydrazone (FCCP) and 5 μM rotenone/antimycin A (R/A) were injected into assay media supplemented with 10 mM glucose and 0.5 mM pyruvate. After each reagent injection, the instrument looped three times with a 3-min mix, 3-min wait and 3-min measurement. The number of cells that remained in each well were counted and used for normalization. Extracellular acidification rate (ECAR) and oxygen consumption rate (OCR) were plotted as a function of time. Replicates (ECAR: *n* = 3. OCR: *n* = 5) were averaged, normalized to cell count, and represented as mean ± STD. All assay kits were purchased from Agilent Seahorse (Santa Clara, CA).

### NMR sample preparation and spectroscopy analysis

All NMR spectra were taken utilizing a Bruker Advance III HD 500 MHz spectrometer with a Prodigy BBO cryoprobe. Where relevant, water suppression was performed with presaturation. Liquid N_2_ flash frozen tumor samples were homogenized using a liquid N_2_-cooled mortar and pestle, removed, weighed and then stored at −80 °C. For the metabolite extraction both homogenized tumor samples and cell pellet samples stored at -80 °C were thawed on ice. Extraction of metabolites was performed using 3 mL of ice-cooled 2:1 methanol: water solution for every 1 x 10^6^ cells and ~ 500 μl of MP Biomedicals lysing matrix D beads. All samples were subjected to 3 freeze-thaw cycles using liquid nitrogen with a 1 min vortex between each cycle to ensure complete homogenization. The homogenates were then centrifuged for 10 min at 4000 × g, supernatant removed, methanol removed through rotary evaporation, lyophilized overnight, and the remaining metabolites were dissolved in D_2_O with 0.5 mM DSS-d_6_, 50 mM K_2_HPO_4_ (pH 7.4). When ^13^C spectra were taken, the [DSS-d_6_] was increased to 10 mM after the acquisition of the ^1^H spectra.

To compare metabolic profiles, ARHI-induced and non-ARHI-induced samples were analyzed by acquiring ^1^H-spectra of the intracellular water-soluble extracted metabolites using 1536 scans, a spectral width (SW) of 10245 Hz and a relaxation delay of 6 s. ^1^H-spectra of the media samples were acquired using 64 instead of 1536 scans. For the ^13^C-glucose feeding study, ^13^C-filtered ^1^H spectra (1D HSQC) for the cell pellet-extracted, re-suspended metabolites were obtained using 8192 scans, a SW of 10245 Hz, 1 increment in F1, and a relaxation delay of 6 s. Finally, for the 5-^13^C-glutamine feeding study ^13^C and ^1^H spectra of the cell pellet-extracted, re-suspended metabolites used, respectively, 2048 and 1024 scans, with a spectral width (SW) of 29760 and 10245 Hz, and a relaxation delay of 6 s. For all of the spectroscopy, DSS-d_6_ was used both as a chemical shift reference (0.00 ppm) and an internal concentration standard for quantification of metabolites. All data were analyzed by Bruker Topspin 2.1. All spectra were manually phased and baseline corrected. The relative concentration of each metabolite was determined by the ratio of the integration of the resonance(s) for each metabolite over the integration value of the DSS-d_6_ internal standard. Each metabolite signal was normalized to the total ^1^H-NMR signal or the viable cell count. Metabolite resonances were identified through reference to either of two online metabolomics databases, HMDB (http://www.hmdb.ca) [38–40], or BMRB (http://www.bmrb.wisc.edu/metabolomics), and when necessary, confirmed by spiking the sample with a known amount of the metabolite in question.

### Quantitation of extracellular ammonia

SKOv3-ARHI cells were grown in 25 mL cell culture flasks and induced with 1ug/mL Dox. The media from each cell sample was harvested and the concentration of ammonia determined using the Quantifluo fluorimetric ammonia assay Kit (BioAssay Systems). The absolute concentration of ammonia in each sample was determined by extrapolation of an NH_4_Cl standard curve after subtracting the fluorescence (λ_Ex_ = 360, λ_Em_ = 450 nm) of the reference media from each sample. Statistical difference was determined by unpaired two tailed *t* test (***, *p* < 0.001) based on 3 replicate samples for each condition.

### [^3^H]-2-deoxyglucose uptake

Cells (2 × 10^5^ cells/well) were plated in 6-well plates and treated with 1ug/mL Dox for 24, 48 or 72 h to induce ARHI expression. The media was removed and 5 μCi of [^3^H]-2-deoxy-D-glucose solution in Krebs Ringer buffer (1.5 mL) was added each well. The cells were incubated for 45 min at 37 °C after which the buffer was aspirated and the cells washed twice with PBS. Adherent cells were trypsinized, transferred to a 15-mL falcon tube, and centrifuged at 500 × g for 10 min. The supernatant was aspirated and the pellet resuspended in 500 μL of sterile PBS. 400 μL of the resuspended pellet was mixed with 1 mL of toluene and 3 mL of scintillation solution in a scintillation vial. The remaining suspension was used for cell counting. The activity of each sample was measured on a scintillation counter and the uptake determined by normalizing the observed counts per minute (cpm) to the number of viable cells. Triplicate samples (*n* = 3) were used for each experimental condition and analysis of the results was performed in GraphPad Prism6.

### 2-DG inhibition studies

SKOv3-ARHI cells (2.5 × 10^4^ cells per well) were grown in 24-well plates with 500 μl McCoy’s 5A (1.5 % glucose) and DMEM (no glucose) media (1:1 ratio). HEY-ARHI cells (2.5 × 10^4^ cells per well) were grown in 24-well plates with 500 mL RPMI-1640 (3 % glucose). The cells were cultured overnight, and then treated with or without Dox (to induce ARHI) and with or without 2-DG (10 mM) for the indicated period of time. Cell viability was assessed with a Sulforhodamine B (SRB) assay. Briefly, 50 μl 30 % trichloroacetic acid (TCA) was added to each well and plates were incubated at 4 °C for 1 h. The plates were rinsed with distilled water and 100 μL of 0.4 % Sulforhodamine B (SRB) in 1 % acetic acid was added to each well. Plates were incubated for 30 min at room temperature, and then rinsed with 1 % acetic acid. SRB was solubilized with 100 μl of 10 mM Tris buffer for 5 min with shaking. Absorbance values were measured on a microplate reader at 570 nm and used to calculate the relative cell viability. Triplicate samples were used for each experimental condition to calculate the mean and standard deviation. Statistical analysis of the results was performed in GraphPad Prism6.

### U-^13^C_6_-glucose feeding study

ARHI was expressed under control of the Tet promoter in SKOv3-ARHI cells and induced with 10 μM Dox for 48 h. The medium was then changed to glucose-depleted SKOv3 medium supplemented with 20 mM U-^13^C_6_-glucose (universal label). 250 μL aliquots of medium were taken at time points of 0, 1, and 8 h to track glucose utilization. After 8 h, the media was removed and cells were trypsinized, counted, pelleted, and extracted. The water-soluble metabolites and medium were analyzed by ^13^C and ^1^H NMR and the integrated values of the metabolite resonances normalized to the viable cell count prior to metabolite extraction. Triplicate samples were used for each experimental condition and analysis of the results was performed in GraphPad Prism6.

### 5-^13^C-glutamine feeding study

SKOv3-ARHI cells were grown under standard conditions and induced with Dox for 24 h. The medium was then changed to glutamine-depleted medium supplemented with 2 mM 5-^13^C-Glutamine (labeled at carbon 5). After 24 h the cells were harvested as described above and analyzed by ^13^C and ^1^H NMR. The integrated values for each metabolite in the ^13^C spectra were normalized to the total integrated ^1^H signal of all water soluble metabolites.

### BPTES inhibition studies

SKOv3-ARHI and HeyA8-ARHI cells were grown in a 96-well assay plates under standard conditions. Each cell line was treated with 10uM, 1uM or 0.1 μM BPTES (in DMSO) or DMSO alone in triplicate samples, for a total of 72 h, and the concentration of DMSO was kept constant throughout. Cells were induced by addition of doxycycline (1 μg/mL) at 72, 48 or 24 h containing each of the BPTES concentrations listed above. Media was changed daily for each sample containing the appropriate additives in the appropriate concentrations. Upon completion of incubation period, media was aspirated and replaced with 100 μL of appropriate fresh media. The Vybrant*®* MTT Cell Proliferation Assay Kit was used to determine cell growth rates in the variable BPTES concentrations at different points of ARHI induction. Briefly, cells were incubated with 1 mM MTT reagent (3-(4,5-dimethylthiazol-2-yl)-2,5-diphenyltetrazolium bromide at 37 °C for 4 h. Each sample was then supplemented with 0.34 mM SDS in 0.1 M HCl and incubated for an additional 5 h. at 37 °C. Each sample was mixed and analyzed in a Synergy H4 microplate reader (Biotek) plate reader at 570 nm. Triplicate samples were used for each experimental condition and analysis of the results was performed in GraphPad Prism6.

### Measurement of NAD+/NADH and ADP/ATP

SKOv3 (parental) or SKOv3-ARHI cells were seeded into a 96-well plate, and treated with 1ug/mL Doxycycline for 12, 24, and 48 h. Cell media was changed every 24 h with the appropriate Dox concentration. The NAD+/NADH-Glo™ Assay kit was obtained from Promega and was carried out based on the instructions to quantify NAD+ and NADH separately. Briefly, after appropriate treatment over the desired time, media was removed and cells were supplemented with 50uL of PBS and 50uL 0.2 N NaOH solution with 1 % DTAB to obtain a cell lysate. To measure NAD+, a 50uL aliquot of cell lysate was treated with 0.4 N HCl and heat quenched at 60 °C for 15 min. Solution was then neutralized with Trizma buffer. NADH samples were heat quenched following the addition of NaOH with 1 % DTAB and solution was neutralized with HCl-Trizma. An equal volume of NAD/NADH-glo Detection Reagent was added to each well with cell lysate, incubated at room temperature for 60 min, and scanned using a luminometer. The ATP/ADP assay kit was also carried out in a 96-well plate with ADP/ATP Ratio Assay kit from Abcam®. Briefly, after Dox treatment, media was removed and each well with cells, including a negative control (no cells), was supplemented with 50uL nucleotide releasing buffer. Each well was then supplemented with 100uL of a reaction mixture containing an ATP monitoring enzyme and scanned in a luminometer to measure ATP levels. Each sample was then administered 10uL of an ADP-converting enzyme and scanned to detect luminescence and determine ADP levels. Seven replicate samples were used for each condition (*n* = 7) and data analysis was performed in GraphPad.

### Uptake of TMRM and H2DCFDA in SKOv3-ARHI

SKOv3-ARHI cells were treated with 1 μg/ml Dox for 24 and 48 h or with 50 nM Rapamycin for 24 h. Cells were then incubated with 20 nM tetramethyl rhodamine methyl ester (TMRM) in FBS-free media for 45 min, washed twice with phosphate buffered saline, and re-suspended in FBS-free media. The mean fluorescence intensity for 10,000 cells was obtained by flow cytometry. In a separate experiment, SKOv3-ARHI and SKOv3 (parental) cells were treated with Dox for 12, 24 and 48 h. The cells were then washed and stained with 5 μM H2DCFDA and analyzed by flow cytometry to obtain the mean fluorescence intensity. Triplicate samples were used for each experimental condition and analysis of the results was performed in GraphPad.

### TOM20 staining for flow cytometry

SKOv3-ARHI cells were seeded into a 75 cm^2^ flask and allowed to reach confluence. Cells were treated with 1 μg/mL of Doxycycline for different time points as previously discussed. After treatment, cells were trypsinized and harvested at 500xg for 10 min. Cells were then fixed in 3 % formaldehyde and permeabilized with 0.1 % Triton X-100. Cells were then counted, aliquoted and incubated in 2 μg of TOM20 antibody (Santa Cruz Biotechnology) for 40 min. Cells were then washed and incubated in F(ab’) 2-Goat anti Rabbit IgH (H + L) Secondary Antibody, Alexa Fluor® 488 conjugate (Thermo Scientific).

### ^1^H-NMR metabolomic analysis of SKOv3-ARHI subcutaneous tumors

Mice bearing subcutaneous SKOv3-ARHI tumors 5-10 mm in diamater were given Dox + sucrose or sucrose alone through drinking water. Groups of four mice were sacrificed at 2 days and 4 days post-induction and their tumors removed and flash frozen in liquid nitrogen. The tumors were homogenized and extracted as described above and analyzed by ^1^H NMR. The integrated signals for each metabolite were normalized to the total integrated ^1^H signal of all water soluble metabolites.

### FDG-PET of SKOv3-ARHI subcutaneous tumors

Subcutaneous SKOv3-ARHI mice were fasted for 3 h, anesthetized using 2 % isoflurane, and injected with 145-180 uCi of [^18^F]-fluorodeoxyglucose (FDG) in 250 μL saline via the tail vein. Each mouse was allowed uptake of [^18^F]-FDG for 30 min followed by a 20 min PET and 10 min CT scan on an Inveon micro PET/CT (Siemens). Following the initial scan (t = 0), mice were given Dox + sucrose or sucrose alone in drinking water and imaged at 2 and 4 days by FDG-PET using the same procedure described above.

### Availability of supporting data

Figures S1-S7 are available in Additional file 1.

## Additional file

Additional file 1: Figure S1. Growth of parental and ARHI-transfected SKOv3 and Hey cells. Effect of Dox treatment on the growth of parental and ARHI-transfected SKOv3 and Hey cells at 24 and 48 h. Western analysis of the effect of ARHI expression on LC3I and LC3II is also presented. **Figure S2.** Western analysis of GLUT1 expression following ARHI induction. **Figure S3.** Analysis of ARHI expression and autophagy markers during Atg5 knockdown. Effect of Atg5 knockdown on LC3I and LC3II levels during ARHI expression in SKOv3-ARHI cells. Immunofluorescence of SKOv3-ARHI cells transfected with GFP-LC3 following ARHI induction with and without Atg5 knockdown. **Figure S4.** Western analysis of LDH and CK expression following ARHI induction. **Figure S5.** Induction of ARHI expression in vivo. Expression of ARHI and LC3 in subcutaneous SKOv3-ARHI tumors at 24-72 h post-treatment with Dox. **Figure S6.** Expression of ACC and Phsopho-ACC by RPPA. **Figure S7.** Fractional ^13^C label incorporation from 5-^13^C-Gln in SKOv3-ARHI. The fractional incorporation of the glutamine ^13^C label into NMR-observable intracellular metabolites following induction of ARHI. (PDF 867 kb)

## Abbreviations

AMPK: 5′ adenosine monophosphate-activated protein kinase
ATG5: Autophagy protein 5
ATP: Adenosine triphosphate
BPTES: Bis-2-(5-phenylacetamido-1,3,4-thiadiazol-2-yl)ethyl sulfide
CK: Choline kinase α
Dox: Doxycycline
DSS: 4,4-dimethyl-4-silapentane-1-sulfonic acid
ECAR: Extracellular acidification rate
FCCP: Carbonyl cyanide-*p*-trifluoromethoxyphenylhydrazone
FDG: [^18^F]-2-fluoro-2-deoxyglucose
FOXO3a: Forkhead box O3 protein
GFP: Green fluorescent protein
GLUT1: Glucose transporter 1
GPC: Glycerophosphocholine
HSQC: Heteronuclear single quantum coherence
Lac: Lactate
LC3: Microtubule-associated proteins 1A/1B light chain 3
LDH: Lactate dehydrogenase
NAG: N-acetyl glutamate
NMR: Nuclear magnetic resonance
OCR: Oxygen consumption rate
Oligo: Oligomycin
PC, PCho: phosphocholine
PET /CT: Positron emission tomography/computed tomography
PPP: Pentose phosphate pathway
R/A: Rotenone/antimycin A
R-5-P: Ribose-5-phsophate
RIP1/RIP3: Receptor-interacting serine/threonine protein kinase1/receptor-interactingserine/threonine protein kinase﻿ 3 complex
ROS: Reactive oxygen species
TCA: Tricarboxylic acid or Krebs cycle
ULK1/2: UNC-51-like kinase 1 and 2
