# Supplementary material for: Induction of autophagy by ARHI (DIRAS3) alters fundamental metabolic pathways in ovarian cancer models
Source: BMC Cancer. 2016 Oct 26;16:824. doi: 10.1186/s12885-016-2850-8 (PMC5080741; doi:10.1186/s12885-016-2850-8)
Supplement: Additional file 1: Figure S1. — Growth of parental and ARHI-transfected SKOv3 and Hey cells. Effect of Dox treatment on the growth of parental and ARHI-transfected SKOv3 and Hey cells at 24 and 48 h. Western analysis of the effect of ARHI expression on LC3I and LC3II is also presented. Figure S2. Western analysis of GLUT1 expression following ARHI induction. Figure S3. Analysis of ARHI expression and autophagy markers during Atg5 knockdown. Effect of Atg5 knockdown on LC3I and LC3II levels during ARHI expression in SKOv3-ARHI cells. Immunofluorescence of SKOv3-ARHI cells transfected with GFP-LC3 following ARHI induction with and without Atg5 knockdown. Figure S4. Western analysis of LDH and CK expression following ARHI induction. Figure S5. Induction of ARHI expression in vivo. Expression of ARHI and LC3 in subcutaneous SKOv3-ARHI tumors at 24-72 h post-treatment with Dox. Figure S6. Expression of ACC and Phsopho-ACC by RPPA. Figure S7. Fractional 13C label incorporation from 5-13C-Gln in SKOv3-ARHI. The fractional incorporation of the glutamine 13C label into NMR-observable intracellular metabolites following induction of ARHI. (PDF 867 kb) [file 12885_2016_2850_MOESM1_ESM.pdf]

Supplemental Data

Induction of autophagy by ARHI (DIRAS3) alters fundamental metabolic pathways in ovarian cancer models

Argentina Ornelas[#1], Christopher R. McCullough[#1], Zhen Lu[#2], Niki M. Zacharias[1,3], Lindsay E. Kelderhouse[1], Joshua Gray[1], Hailing Yang[2], Brian J. Engel[1], Yan Wang[2], Weiqun Mao[2], Margie N. Sutton[2], Pratip K. Bhattacharya[1], Robert C. Bast Jr.[2], Steven W. Millward[*1,3]

1. Department of Cancer Systems Imaging, the University of Texas M.D. Anderson Cancer Center
2. Department of Experimental Therapeutics, the University of Texas M.D. Anderson Cancer Center
3. Department of Bioengineering, Rice University.

Authors shared equally in this work. * Corresponding Author.

Email: Argentina Ornelas - AOrnelas@mdanderson.org, Christopher R. McCullough - CRMcCullough@mdanderson.org, Zhen Lu - zlu@mdanderson.org, Niki M. Zacharias - NMZacharias@mdanderson.org, Lindsay E. Kelderhouse - LEKelderhouse@mdanderson.org, Joshua Gray - JPGray@mdanderson.org, Hailing Yang – Hyang3@mdanderson.org, Brian Engel - BJEngel@mdanderson.org, Yan Wang - yanwang@mdanderson.org, Weiqun Mao - WMao@mdanderson.org, Margie N. Sutton - MNSutton@mdanderson.org, Pratip K. Bhattacharya - PKBhattacharya@mdanderson.org, Robert C. Bast Jr. - rbast@mdanderson.org, Steven W. Millward - smillward@mdanderson.org.

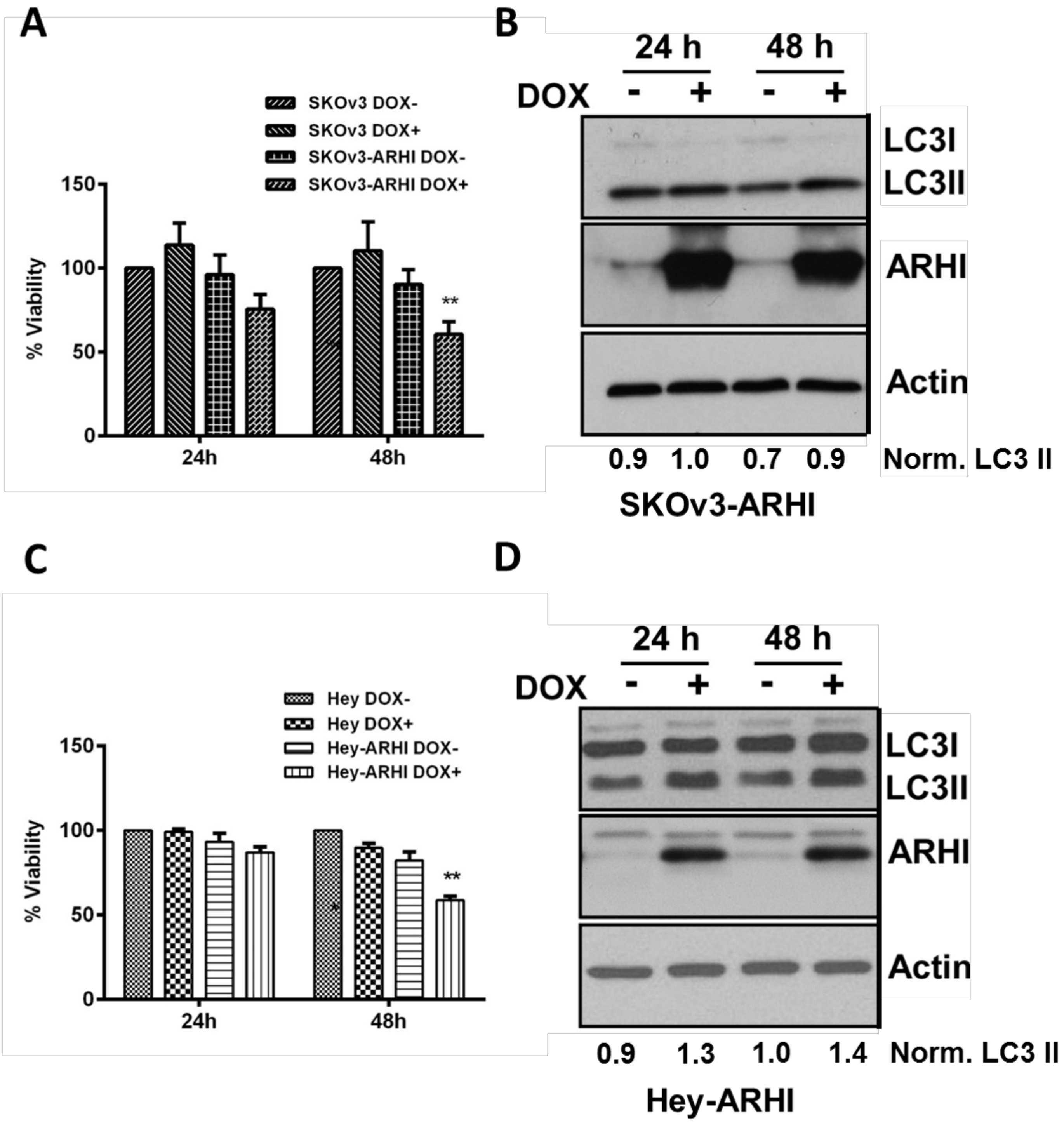

Supplemental Figure S1: Growth of parental and ARHI-transfected SKOv3 and Hey cells. A) SKOv3 and SKOv3-ARHI or **C)** Hey and Hey-ARHI cells were grown in the presence and absence of Dox (1 μg/mL) for 24 and 48 hours. Both SKOv3-ARHI and Hey-ARHI cells showed inhibition of growth at 48 hours in the presence of Dox while the parental lines showed no change. Triplicate samples were used to calculate the mean and standard deviation. Statistical analysis was obtained using a two-tailed t-test in GraphPad. (**, $p < 0.01$). **B)** SKOv3-ARHI and **D)** Hey-ARHI cells were treated with Dox (1 μg/mL) for 24 or 48 hours, lysed and analyzed by western blotting with antibodies to LC3 and ARHI. The LC3 II band intensities were measured in ImageJ and normalized to the corresponding actin band intensity to obtain the normalized LC3II level for each condition (shown at the bottom of each panel). Expression of ARHI was accompanied by an increase in normalized LC3 II levels indicating induction of autophagy.

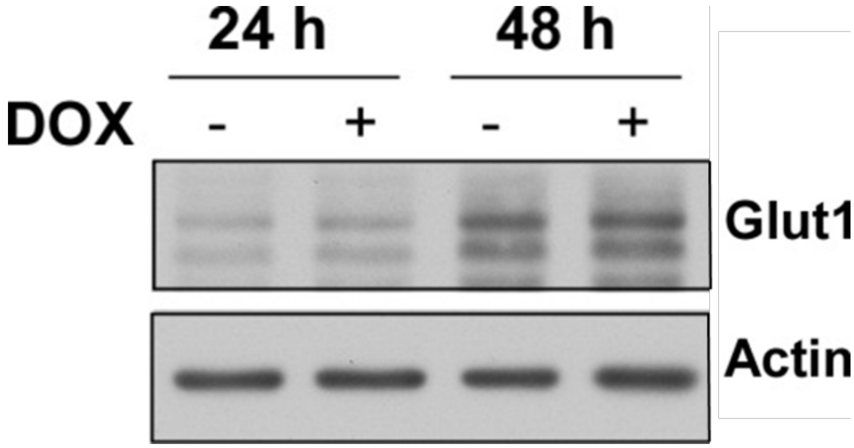

Supplemental Figure S2: Western analysis of GLUT1 expression following ARHI induction. SKOv3-ARHI cells were treated with and without Dox for 24 and 48 hrs and the cell lysates were subjected to Western analysis GLUT1 antibody.

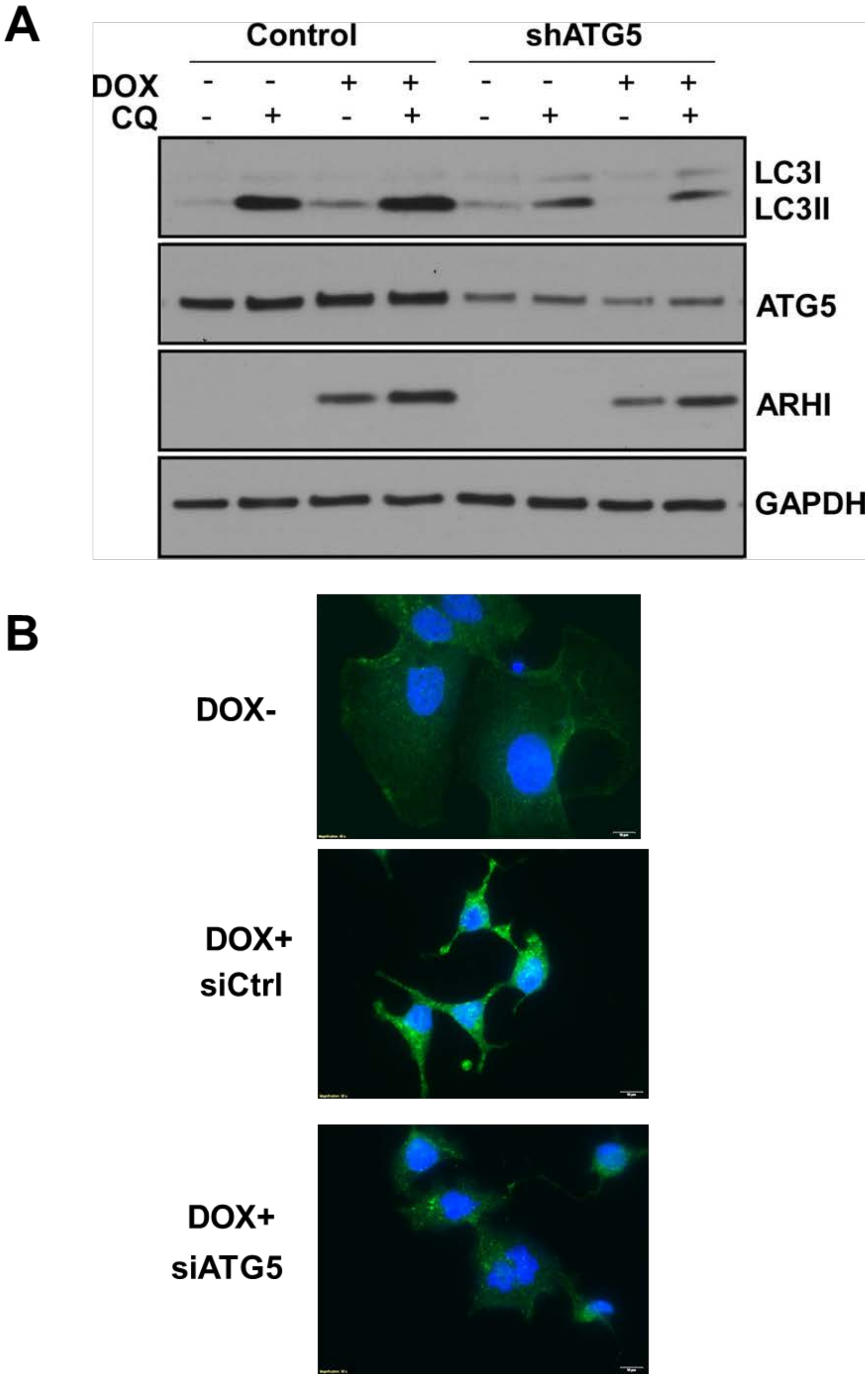

Supplemental Figure S3: A) Western analysis of ARHI expression and autophagy markers during Atg5 knockdown. SKOv3-ARHI-shCtrl and SKOv3-ARHI-shATG5 cells were treated with and without Dox and chloroquine (CQ) for 48 hrs and the cell lysates were subjected to Western analysis with ATG5, ARHI, and LC3 antibodies. Addition of DOX results in increased expression of ARHI and an increase in LC3II indicating induction of autophagy. ATG5 knockdown results in decreased LC3II and increased LC3I indicating inhibition of autophagy. Treatment with chloroquine results in an accumulation of LC3II which is attenuated by Atg5 knockdown, confirming that expression of ARHI increases autophagic flux. **B)** SKOv3-ARHI cells stably transfected with LC3-GFP (green) were cultured in the presence or absence of Dox and transiently transfected with either control (SiCtrl) or Atg5-targeted (siATG5) siRNA. Atg5 knockdown resulted in almost complete elimination of fluorescent LC3 puncta by fluorescence microscopy.

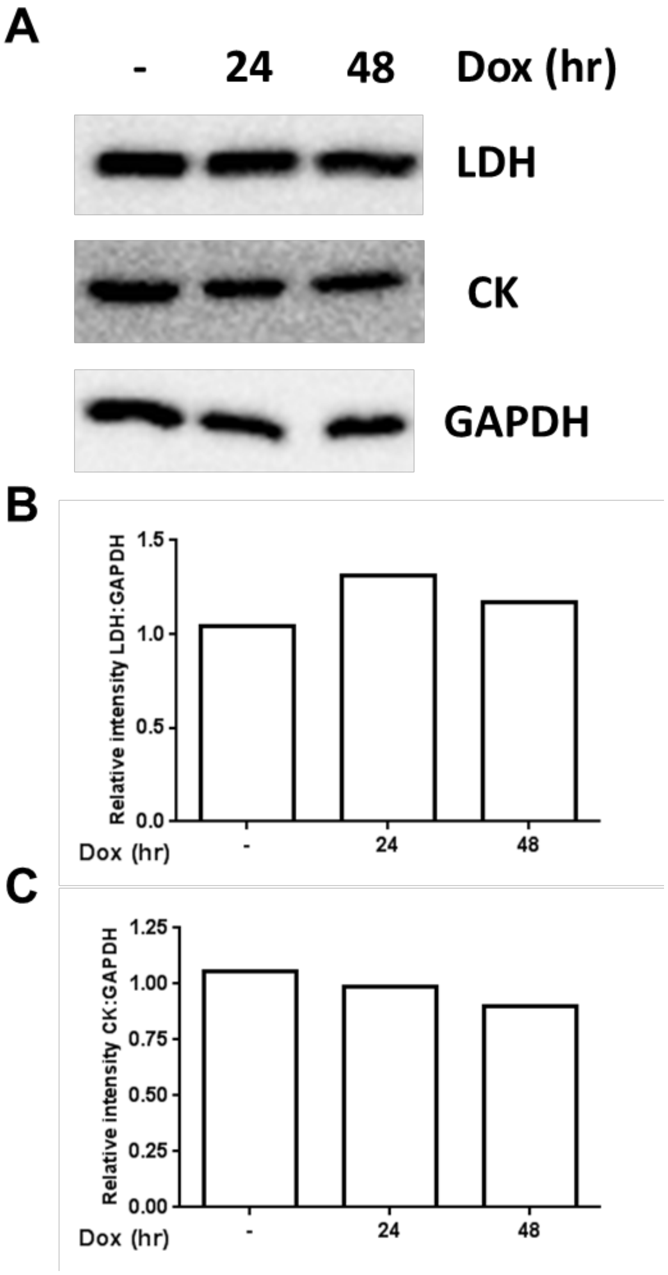

Supplemental Figure S4: A) Western blot analysis shows that ARHI expression results in increased expression of lactate dehydrogenase (LDH) by approximately 20% at 24 hours, while expression of choline kinase α (CK) is reduced by approximately 15% by 48 hours. The intensities of the LDH **B)** and CK **C)** bands in the western blot were quantitated in ImageJ and normalized to the intensity of the corresponding GAPDH band.

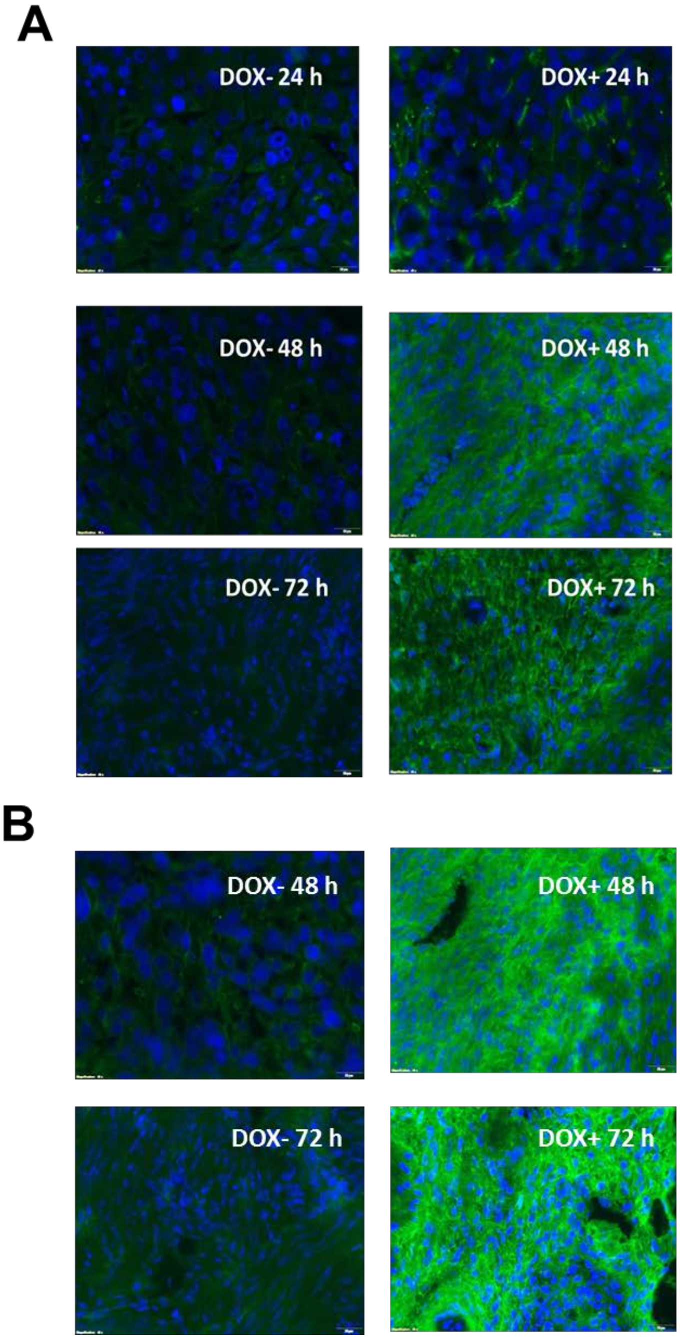

Supplemental Figure S5: Induction of ARHI expression *in vivo*. A) SKOv3-ARHI cells were implanted subcutaneously and allowed to grow to 5-10 mm in diameter. Mice were then placed on drinking water containing Dox + sucrose (Dox+) or sucrose only (Dox-). At the indicated time points the tumor was removed, sectioned, and stained for ARHI expression. At 24 hours post-induction ARHI expression is only slightly above background while at 48 hours it is significantly higher. **B)** Tumor sections were stained for LC3 expression at 48 and 72 hours following Dox treatment. LC3 expression was robustly induced relative to control tumors at each time point.

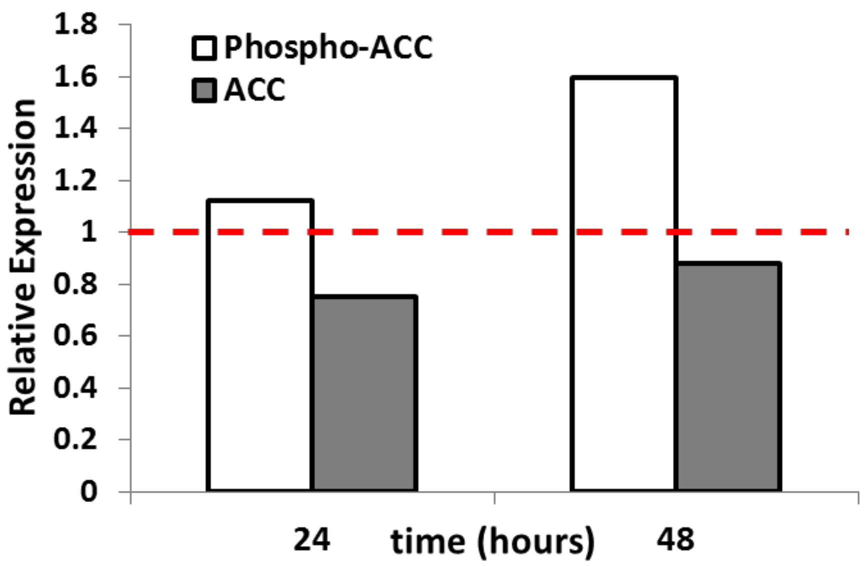

Supplemental Figure S6: Expression of ACC and Phospho-ACC by RPPA. The levels of ACC and phospho-ACC (Ser79) in SKOv3-ARHI cell lysates were determined by Reverse Phase Protein Array (RPPA) [1]. The relative expression was determined by normalizing the signal observed at 24 and 48 hrs post-induction to the signal observed in control non-induced SKOv3-ARHI lysates (dotted red line).

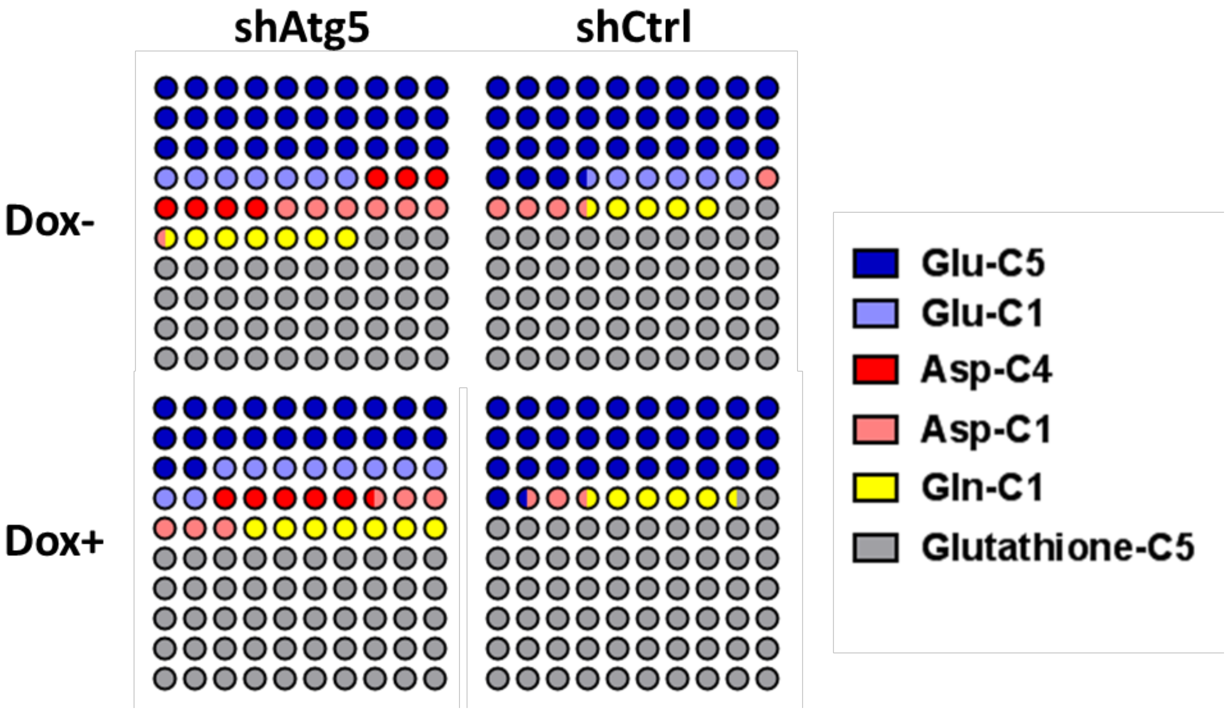

Supplemental Figure S7: Fractional ^{13}C label Incorporation from 5-^{13}C-Gln in SKOv3-ARHI. Fractional label incorporation of ^{13}C in intracellular metabolites between 24 and 48 hours in culture. Values were calculated by dividing the ^{13}C signal for each metabolite by the sum of the intracellular water-soluble ^{13}C signals.

References

1. Iadevaia S, Lu Y, Morales FC, Mills GB, Ram PT. Identification of optimal drug combinations targeting cellular networks: integrating phospho-proteomics and computational network analysis. Cancer research. 2010;70(17):6704-14. doi:10.1158/0008-5472.CAN-10-0460.
